# Examination of the relationship between the empathic tendencies and sleep quality of caregivers of cancer patients

**DOI:** 10.1007/s00520-025-09255-6

**Published:** 2025-02-21

**Authors:** Müjde Kerkez, Muhammet Faruk Yiğit, Zeynep Yaztürk

**Affiliations:** 1 Faculty of Health Sciences, Department of Nursing, Şırnak University, Mehmet Emin Acar Campus, Yeni Neighbourd, Cizre Street, Şırnak, Türkiye; 2 Faculty of Health Sciences, Department of Nursing, Van Yüzüncü Yıl University, Van, Türkiye; 3 Ankara Directorate of Public Health Services, Ankara, Türkiye

**Keywords:** Caregivers, Empathy, Empathic tendency, Sleep quality, Quality of care

## Abstract

**Purpose:**

The present study aims to reveal the relationship between the empathic tendencies and sleep quality of caregivers of cancer patients.

**Material and method:**

This cross-sectional study was conducted with caregivers of cancer patients registered in the oncology unit of a hospital between May and July 2024 (*n* = 346). The study data were collected using a sociodemographic information form, the Empathic Tendency Scale, and the Pittsburgh Sleep Quality Index. The data were analyzed using the Pearson correlation and hierarchical regression analyses as well as descriptive statistics.

Findings.

59.5% of the participants were female and 88.2% lived with patients. 62.7% of the cancer patients were male and 61.1% were partially dependent in daily life activities. The caregivers had a mean total score of 70.58 ± 16.85 on the Empathic Tendency Scale and a mean total score of 4.40 ± 3.94 on the Pittsburgh Sleep Quality Index. It was found that there was a negative, strong, and statistically significant relationship between the mean total scores on the Empathic Tendency Scale and the Pittsburgh Sleep Quality Index (*r* = − 0.924; *p* < 0.001). According to the hierarchical regression model, certain sociodemographic features of the caregivers explained the variance in empathic tendency (Adj. *R*^2^ = 0.607; *p* < 0.001), whereas when sleep quality components were added to the model, the variance in empathic tendency was explained (Adj. *R*^2^ = 0.896; *p* < 0.001).

**Conclusion:**

The caregivers of cancer patients were found to have high empathic tendencies, which was associated with high sleep quality. Furthermore, low empathic tendencies among the caregivers were found to be primarily associated with increasing length of care and advancing patient age. The findings underscore the pivotal role that empathic approaches play in enhancing the quality of care, underscoring the necessity for augmented interventions that prioritize a compassionate approach, and the management of empathy in caregivers of cancer patients.

## Introduction

Cancer remains a major public health issue with an increasing prevalence worldwide [1]. According to global cancer statistics, more than 20 million individuals were newly diagnosed with cancer in 2022, and this number is projected to exceed 35 million by the year 2050 [2]. According to a report by the World Health Organization (WHO), significant global inequalities in cancer and its treatments persist, and the current health systems of some countries are unable to meet all relevant needs [3]. Therefore, the burden of cancer care is increasingly shifting towards non-official caregivers. Current evidence suggests that intensive and episodic cancer care has a profound impact on the well-being and quality of life of caregivers [4, 5].

Caregivers often serve as the primary caregivers of cancer patients, typically providing 24/7 care. The provision of this care encompasses all aspects of the caregiver’s physical, psychological, social, and economic life [6]. The effects of the care process on caregivers range from increased spiritual well-being, therapeutic communication, and personal growth to significant changes such as care burden, social isolation, loss of self-identity, sleep deprivation, and economic difficulties [7, 8]. Current evidence suggests that symptoms of insomnia are a common problem among caregivers [9, 10].

Sleep plays a vital role in terms of physical and psychological health. Sleep deprivation can lead to serious cognitive and physical health problems, as well as depression, low coping mechanisms, diminished self-efficacy, and decreased levels of optimism among caregivers [11, 12]. Moreover, low coping mechanisms refer to an individual’s inability to develop or apply effective and healthy strategies to cope with stress and often lead to the use of dysfunctional or maladaptive coping strategies [13]. For example, poor sleep quality increases the likelihood that individuals perceive daily life events (e.g., interpersonal relationships and work-related responsibilities) as stressors rather than harmless events, increasing the risk of resorting to unhealthy behaviors (e.g., overeating) to cope with stress [14]. A recent systematic review on caregivers of patients with cancer (*n* = 10) reported that at least 72% of the caregivers experienced moderate to severe sleep disorders [15]. Therefore, it is clear that caregivers require assistance, support, and, most importantly, interventions to mitigate these negative consequences [16]. Understanding the experiences of caregivers is central to their empowerment and sustainability of care. Supporting caregivers is fundamental to palliative care and is reflected in the quality of care provided to the patient [17]. Recent studies have focused on increasing caregivers’ levels of empathy to improve the quality of life of both them and their care recipients [17, 18].

Empathy is a complex bio-psycho-social concept consisting of at least two categories, namely cognitive and emotional [19]. Among these, emotional empathy is referred to as empathic tendency and signifies the acceptance or sharing of an individual’s emotional state through a mutual experience [20]. Empathic tendencies improve overall quality of life, reinforce social support networks, reduce feelings of loneliness, and strengthen coping mechanisms against stress [21]. Current evidence also shows that empathy plays a vital role in health care and predicts a higher quality of care for patients [20, 21]. However, while empathy is highlighted as being beneficial in many respects, these outcomes may not always be positive for the empath [17, 22]. In the related literature, the effect of empathy on caregivers is particularly contradictory and the number of studies on this issue is limited. For example, it has been reported that higher levels of empathy in caregivers are significantly associated with reduced levels of depression, burden, and stress [23, 24]. On the other hand, it has been emphasized that when caregivers with high emotional empathy intensely share the distress of the person they care for, it becomes difficult for them to cope with their own emotional burden and their lives may be negatively affected [17, 22].

A growing body of research emphasizes the critical role of sleep on emotional processing and empathy. Sleep deprivation may impair the functionality of brain regions responsible for emotional information processing, such as the amygdala and prefrontal cortex, negatively affecting the ability to recognize emotions and empathize [25–27]. This may lead to an increase in negative emotional reactions and a decrease in empathic sensitivity. Guadagni et al. found that poor sleep quality negatively affects both parallel and reactive components of empathy and reduces activity in brain regions associated with empathy. For example, fMRI studies have shown that reduced activity in the anterior insula and anterior cingulate cortex was observed in individuals with poor sleep quality [28].

The relationship between empathy and sleep quality among caregivers is an area that has not been sufficiently explored in the literature. Studies on various populations have shown that inadequate sleep exacerbates stress responses, impairs emotional regulation, and reduces empathic capacity [29]. Furthermore, most studies on caregivers have focused on caregivers of patients with neurodegenerative disorders and have not examined the effects of empathy and well-being on cancer caregivers. In this context, there is a significant gap in the literature regarding the relationship between empathy levels and sleep quality in cancer caregivers. This study hypothesizes that empathic tendencies are significantly associated with sleep quality among caregivers of cancer patients and that sociodemographic factors such as age, gender, and education level modulate empathic tendencies and their influence on sleep quality.

## Material and method

This cross-sectional study was conducted between May and July 2024 with the caregivers of patients registered in Van Yüzüncü Yıl University Dursun Odabaş Medical Center Medical Oncology and Radiation Oncology units. The study sample was calculated using the G Power software. As a result of the calculations made with parameters of medium effect size (f^2^ = 0.13), 95% power (1-β = 0.95), and 95% confidence interval (α = 0.05), it was determined that it was necessary to reach at least 242 caregivers [25, 30]. The participants were selected using the random sampling method. Before the questionnaires were distributed, the participants were given detailed information about the purpose and methodology of the study. Those who agreed to participate in the study signed the informed consent form. Throughout the study, care was taken to protect the confidentiality and privacy of the participants and it was guaranteed that personal information would not be shared with third parties. No personal information was requested to be included in the questionnaire forms. Each participant was given 7–10 min to complete the questionnaires. At the end of the allotted time, they were asked to return the forms to the researcher. The questionnaire was distributed over a period of 2 months. Caregivers who (i) were 18 years of age or older, (ii) had been providing care to a cancer patient for at least 6 months, (iii) could read and write, (v) had no communicative and mental problems, and (vi) agreed to participate were included in the study. Caregivers who provided paid care services and who had any psychiatric disability were excluded. In this direction, the study questionnaire was completed and analyzed with a total of 346 caregivers.

### Data collection methods and tools

The study data were collected using a sociodemographic information form, the Empathic Tendency Scale (ETS), and the Pittsburgh Sleep Quality Index (PSQI).

#### Sociodemographic information form

This form was prepared by the researchers based on the related literature [11, 31]. The form includes 15 questions on the sociodemographic features of the caregivers (gender, age, marital status, parental status, educational status, perceived economic status, tobacco use, tea/coffee consumption, presence of chronic disease, relation to the patient, duration of care) and 4 questions on the care recipients (age, gender, diagnosis, degree of dependency in daily life activities).

#### Empathic Tendency Scale (ETS)

The five-point Likert-type scale developed by Dökmen [20], consisting of 20 items and two sub-dimensions (positive–negative), is designed to measure the emotional sensitivity of individuals in the face of personal events. The minimum score on the scale is 20 and the maximum score is 100. The scale is scored by considering positive and negative items. Items 3, 6, 7, 8, 11, 12, 13, and 15 in the scale are negative questions and are reverse coded. Empathic tendency scores are grouped as low (45–67), moderate (68–78), and high (79–95). Higher scores on the scale indicate higher levels of empathic tendency. The Cronbach’s alpha internal consistency coefficient of the scale is 0.86. In the present study, the Cronbach’s alpha internal consistency coefficient was calculated as 0.91.

#### Pittsburgh Sleep Quality Index (PSQI)

The Turkish validity and reliability study of the scale developed by Buysse et al. was conducted by Aǧargün et al. [32]. The PSQI, which evaluates sleep quality over a period of 1 month, includes a total of 24 questions. Nineteen of these are self-report questions and are answered by the patient, while five questions are answered by the spouse of the respondent or a housemate and are used only for clinical information without being included in the scoring. The last of the self-report questions (question 19) is about the presence or absence of a housemate or spouse and is not used in the scoring process. The 18 items included in the scoring are grouped into seven component scores. These components are subjective sleep quality, sleep latency, sleep duration, habitual sleep activity, sleep disorder, sleep medication use, and daytime dysfunction. Each item is scored on a scale of 0–3 points and the sum of the seven component scores yields the total PSQI score. The total score ranges from 0 to 21. A total score higher than 5 indicates poor sleep quality. The Cronbach’s alpha internal consistency coefficient of the scale is 0.80. In the present study, this coefficient was calculated as 0.77.

### Data analysis

The study data were evaluated using the SPSS 25.0 package program. Mean, number, percentage, and standard deviation were used to present descriptive characteristics. The data that were found to be normally distributed according to the Kolmogorov–Smirnov Z test were tested with the Pearson correlation and hierarchical regression analyses.

The Pearson correlation analysis was used to determine the direction and strength of the linear relationship between the independent variables and the dependent variable. Additionally, the hierarchical linear regression analysis was also performed to examine the effects of the independent variables on the empathic tendency total score in more detail and to check the effects of other variables. This analysis was used to assess the overall explanatory power of the model and the significance levels of the independent variables on the dependent variable [28, 29]. Prior to the hierarchical linear regression analysis, the variables to be included in the model were evaluated using multiple linear regression. In this analysis, first, linearity was ensured by examining the correlations between the Empathic Tendency Scale, the sociodemographic information form, and the PSQI. The variables found to be significant (*p* < 0.05) were subjected to multiple linear regression. As a result of the regression, multicollinearity problems were eliminated by removing the variables with high VIF values throughout the Emphatic Tendency Scale (*p* < 0.05).

In the final model, the patient’s age, educational status, economic status, status of living with the patient, presence of other dependents (both the sick person and other household members, such as children or the elderly, within the same household) and duration of care, and subcomponents of the PDQI, namely the categorized variables of sleep latency, sleep duration, habitual sleep activity, sleep disorder, and use of sleep medication, were used as independent variables. The overall significance level of the model was evaluated with *p*-value while the explanatory power of the model was determined with *R*^2^ and Adj. *R*^2^ values. The level of statistical significance was accepted as *p* < 0.05.

### Declaration of ethics

For the present study, approval was obtained from Van Yüzüncü Yıl University Non-Interventional Clinical Research Ethics Committee (2024/03–20) as well as the institution where the study was conducted (2024/ E-54355720–800–548002). During the study, the rules specified in the Declaration of Helsinki were adhered to and written informed consent was obtained from the patients participating in the study.

## Findings

In this section, the sociodemographic features of the participants and the results of the analyses related to the Empathic Tendency Scale and the Pittsburgh Sleep Quality Index are presented.

Table 1 shows that 59.5% of the participants were female, 69.9% were married, 37.3% were the spouse of the care recipient, and 88.2% lived with the patient. Of the caregivers, 30.1% had children, 37.3% were high school graduates, and 65.0% were unemployed. 41.9% perceived their economic situation as poor, while 59.9% stated that they did not have any chronic disease and did not use regular medication. 49.1% of the caregivers stated that they provided personal hygiene and general care for the care recipient and 32.4% stated that the duration of care was between 0 and 1 year. 77.7% of the participants had other dependents (both the sick person and other household members, such as children or the elderly, within the same household) and 65.0% had no experience in caregiving. 59.5% did not smoke and 74.0% did not consume caffeine (Table 1). 42.2% had an ETS score ≥ 79–95 and 43.6% had an ETS score ≤ 45–67. In other words, about half of the caregivers show a strong tendency to understand the emotions of others and to respond empathically, while the other caregivers have lower empathic tendencies. Furthermore, considering that the scores are distributed in this range, it can be said that there is a significant heterogeneity in empathy levels among caregivers. 57.8% had a PSQI score ≤ 5 and 42.2% had a PSQI score > 5 (Table 1). In other words, more than half of the caregivers exhibited good sleep quality.

**Table 1:** Sociodemographic features of the caregivers

		Frequency	Percentage
Age		40.57 ± 15.84	
Gender	Female	206	59.5
	Male	140	40.5
Marital status	Married	242	69.9
	Single	104	30.1
Parental status	Yes	104	30.1
	No	242	69.9
Educational status	Literate	36	10.4
	Primary education	94	27.2
	High school	129	37.3
	Associate degree	75	21.7
	License	12	3.5
Employment status	Employed	121	35.0
	Unemployed	225	65.0
Perceived economic status	Poor	145	41.9
	Average	140	40.5
	Well	61	17.6
Chronic disease status	Yes	142	41.0
	No	204	59.0
Regular medication use	Yes	142	41.0
	No	204	59.0
Relation to the patient	Spouse	129	37.3
	Daughter	67	19.4
	Son	109	31.5
	Other	41	11.8
Status of living with the patient	Yes	305	88.2
	No	41	11.8
Presence of other dependents	Yes	269	77.7
	No	77	22.3
Duration of care	0–1 year	112	32.4
	1–2 years	106	30.6
	2–3 years	78	22.5
	3–4 years	18	5.2
	Over 4 years	32	9.2
Status of experience in caregiving	Yes	225	65.0
	No	121	35.0
Caregiving behaviors	Hospital operations	124	35.8
	Medication intake	52	15.0
	Personal hygiene and care	170	49.1
Tobacco use	Yes	140	40.5
	No	206	59.5
Caffeine consumption	Yes	90	26.0
	No	256	74.0
ETS Global Score	ETS ≤ 45–67	151	43.6
	ETS = 68–78	49	14.2
	ETS ≥ 79–95	146	42.2
PSQI Global Score	PSQI ≤ 5	200	57.8
	PSQI > 5	146	42.2
	Total	346	100.0

*PSQI* Pittsburgh Sleep Quality Index (PSQI ≤ 5: good sleep quality; PSQI > 5: poor sleep quality), *ETS* Emphatic Tendency Scale (ETS ≤ 45–67: low emphatic tendency; ETS = 68–78: moderate emphatic tendency; ETS ≥ 79–95: high emphatic tendency)

According to Table 2, the mean age of the care recipients was 63.41 ± 6.47 years, 62.7% were male, 22.5% were diagnosed with lung cancer, and 61.1% were partially dependent in daily life activities (Table 2).

**Table 2:** Demographic distribution of the care recipients

		Frequency	Percentage
Age		63.41 ± 6.47	
Gender	Female	129	37.3
	Male	217	62.7
Diagnosis	Lung cancer	78	22.5
	Breast cancer	55	15.9
	Stomach cancer	51	14.7
	Colon cancer	48	13.9
	Brain tumor	26	7.5
	Prostate cancer	18	5.2
	Bladder cancer	16	4.6
	Esophageal cancer	13	3.8
	Pancreatic cancer	12	3.5
	Other^a^	29	8.4
Degree of dependency	Completely dependent	73	21.1
	Partially dependent	211	61.0
	Fully independent	62	17.9
	Total	346	100.0

^a^Kidney, liver, cervix, uterus, lymphoma cancers

Table 3 shows the participants’ mean scores on the Emphatic Tendency Scale (ETS) and the Pittsburgh Sleep Quality Index (PSQI) and their subscales. The mean ETS total score of the participants was 70.58 ± 16.85 and the mean PSQI total score was 4.40 ± 3.94. The mean scores of the caregivers on the ETS subscales were 45.35 ± 10.47 for positive empathic tendency and 25.22 ± 6.94 for negative empathic tendency, respectively. The results indicate that participants generally exhibited moderate levels of empathic tendencies, with a stronger inclination towards positive empathic responses such as understanding and compassion. This suggests that caregivers maintain moderate to high empathy levels that promote effective emotional connections. Additionally, the mean scores on the PSQI subscales were 1.49 ± 1.32 for subjective sleep quality, 0.60 ± 0.77 for sleep latency, 0.03 ± 0.19 for sleep duration, 0.28 ± 0.58 for habitual sleep activity, 0.16 ± 0.36 for sleep disorder, and 1.53 ± 1.29 for sleep medication use, respectively. The results showed that more than half of the caregivers slept relatively well. Subscale analyses revealed that caregivers perceived their subjective sleep quality as satisfactory, experienced slight delays in sleep onset, and maintained consistent sleep routines with minimal disturbance (Table 3).

**Table 3:** Mean scores of the caregivers on the Emphatic Tendency Scale and Pittsburgh Sleep Quality Index

	Minimum	Maximum	Mean	Std. deviation
**ETS total**	20	100	70.58	16.85
Positive empathic tendency	12.00	60.00	45.35	10.47
Negative empathic tendency	8.00	40.00	25.22	6.94
**PSQI total**	0.00	21.00	4.40	3.94
Subjective sleep quality	0.00	3.00	1.49	1.32
Sleep latency	0.00	3.00	0.60	0.77
Sleep duration	0.00	3.00	0.03	0.19
Habitual sleep activity	0.00	3.00	0.28	0.58
Sleep disorder	0.00	3.00	0.16	0.36
Sleep medication use	0.00	3.00	1.53	1.29

*ETS* Emphatic Tendency Scale, *PSQI* Pittsburgh Sleep Quality Index

Table 4 shows the correlations between the Emphatic Tendency Scale (ETS) and Pittsburgh Sleep Quality Index (PSQI) and their subscales. It was found that there was a negative, very strong, and statistically significant relationship between the mean total scores on the ETS and the PSQI (*r* = − 0.924; *p* < 0.001). This suggests that caregivers with higher empathic tendencies experience better overall sleep quality, emphasizing the relationship between emotional engagement and physical well-being. Furthermore, negative, very strong, and statistically significant relationships were also observed between the mean ETS total scores and the mean scores on the PSQI subscales of subjective sleep quality, sleep latency, and daytime dysfunction (*r* = − 0.938; *p* < 0.001; *r* = − 0.802, *p* < 0.001; *r* = − 0.887, *p* < 0.001, respectively). These findings highlight the significance of addressing emotional and physical well-being to support caregivers effectively (Table 4).

**Table 4:** Correlation analysis results between scales

		1	2	3	4	5	6	7	8	9	10	11
ETS		1										
Positive empathic tendency	*r*	.979^**^	1									
	*p*	< 0.001										
Negative empathic tendency	*r*	.951^**^	.867^**^	1								
	*p*	< 0.001	< 0.001									
PSQI	*r*	− .924^**^	− .940^**^	− .824^**^	1							
	*p*	< 0.001	< 0.001	< 0.001								
Subjective sleep quality	*r*	− .938^**^	− .935^**^	− .867^**^	.964^**^	1						
	*p*	< 0.001	< 0.001	< 0.001	< 0.001							
Sleep latency	*r*	− .802^**^	− .842^**^	− .677^**^	.840^**^	.797^**^	1					
	*p*	< 0.001	< 0.001	< 0.001	< 0.001	< 0.001						
Sleep duration	*r*	− .272^**^	− .206^**^	− .348^**^	.280^**^	.225^**^	0.101	1				
	*p*	< 0.001	< 0.001	< 0.001	< 0.001	< 0.001	0.061					
Habitual sleep activity	*r*	− .484^**^	− .521^**^	− .390^**^	.539^**^	.386^**^	.392^**^	− 0.095	1			
	*p*	< 0.001	< 0.001	< 0.001	< 0.001	< 0.001	< 0.001	0.078				
Sleep disorder	*r*	− .313^**^	− .271^**^	− .351^**^	.390^**^	.394^**^	− 0.066	.308^**^	.204^**^	1		
	*p*	< 0.001	< 0.001	< 0.001	< 0.001	< 0.001	0.224	< 0.001	< 0.001			
Sleep medication use	*r*	− .413^**^	− .439^**^	− .340^**^	.574^**^	.500^**^	.517^**^	.450^**^	.112^*^	0.080	1	
	*p*	< 0.001	< 0.001	< 0.001	< 0.001	< 0.001	< 0.001	< 0.001	0.038	0.136		
Daytime dysfunction	*r*	− .887^**^	− .918^**^	− .769^**^	.972^**^	.948^**^	.827^**^	.224^**^	.472^**^	.314^**^	.498^**^	1
	*p*	< 0.001	< 0.001	< 0.001	< 0.001	< 0.001	< 0.001	< 0.001	< 0.001	< 0.001	< 0.001	

^**^p < 0.001; *p < 0.05; *ETS* Emphatic Tendency Scale, *PSQI* Pittsburgh Sleep Quality Index, *r* Pearson correlation

Negative, weak, but statistically significant relationships were also found between the mean scores of ETS total and the subscales of sleep duration and sleep disorder (*r* = − 0.272, *p* < 0.001; *r* = − 0.313, *p* < 0.001). Negative, weak, but statistically significant relationships were found between the mean scores of ETS total and the subscales of habitual sleep efficacy and sleep medication use (*r* = − 0.484, *p* < 0.001; *r* = − 0.413, *p* < 0.001) (Table 4).

Positive and statistically significant relationships were found between the ETS total mean score and the subscales of positive and negative empathic tendency as well as between the total scores on the PSQI and its subscales (*p* < 0.001). These findings suggest that the empathic tendencies of the caregivers have a significant effect on their sleep quality (Table 4).

Table 5 shows the correlation between the Emphatic Tendency Scale and some demographic characteristics of the caregivers. A statistically significant, positive, and moderate correlation was found between the Emphatic Tendency Scale and the educational status variable as well as the presence of other dependents (*r* = 0.508, *p* < 0.001; *r* = 0.523, *p* < 0.001, respectively). This finding suggests that caregivers’ empathic tendencies become more pronounced when caring for more than one person in the same household. Empathy may play an important role in managing multiple caregiving responsibilities. There was a moderate, statistically significant, and negative relationship between the participants’ mean Emphatic Tendency Scale score, age, and duration of care (*r* = − 0.562, *p* < 0.001; *r* = − 0.624, *p* < 0.001, respectively). Additionally, a statistically significant relationship was found between the participants’ mean Emphatic Tendency Scale score, perceived economic status, and the status of living with the patient (*r* = 0.289, *p* < 0.001; *r* = 0.334, *p* < 0.001, respectively) (Table 5).

**Table 5:** Correlation results between the Emphatic Tendency Scale and some demographic variables

			1	2	3	4	5	6	7
1	ETS	*r*	1						
		*p*							
2	Age	*r*	− .562^**^	1					
		*p*	< 0.001						
3	Educational status	*r*	.508^**^	− .328^**^	1				
		*p*	< 0.001	< 0.001					
4	Perceived economic status	*r*	.289^**^	− .245^**^	0.003	1			
		*p*	< 0.001	< 0.001	0.957				
5	Status of living with the patient	*r*	.334^**^	.150^**^	.436^**^	0.060	1		
		*p*	< 0.001	< 0.001	< 0.001	0.262			
6	Presence of other dependents	*r*	.523^**^	− .633^**^	.318^**^	.443^**^	− .196^**^	1	
		*p*	< 0.001	< 0.001	< 0.001	< 0.001	< 0.001		
7	Duration of care	*r*	− .624^**^	.559^**^	− .593^**^	− .325^**^	− .121^*^	− .508^**^	1
		*p*	< 0.001	< 0.001	< 0.001	< 0.001	0.024	< 0.001	

^**^p < 0.001; *p < 0.05; *ETS* Emphatic Tendency Scale, *r* Pearson correlation

According to Table 6, model 1, which includes the patient’s age, education level, perceived income, status of living with the patient, presence of other dependents, and duration of care, explained 60.7% of the variance in empathic tendency among the participants. The addition of components of sleep quality to this model explained an additional 28.9% of the variation in empathic tendency. However, the variables of sleep duration, sleep disorder, and sleep medication use among the components of PSQI were not statistically significant predictors in the model (*p* > 0.05), whereas sleep latency and habitual sleep activity were significant predictors (*p* < 0.05). Furthermore, model 1 showed that living with the patient (*B* = 21.968, *β* = 0.422, *p* < 0.001) and the presence of other dependents (*B* = 12.163,* β* = 0.301, *p* < 0.001) had a significant positive effect on empathic tendencies. However, in model 2, after adding sleep-related variables, the presence of other dependents turned negative (*B* = − 5.274, *β* = − 0.130, *p* < 0.001), suggesting that caregiving burden combined with sleep-related stress negatively impacts empathy. Sleep duration (*B* = − 8.825, *β* = − 0.076, *p* = 0.019) and sleep disturbances (*B* = 1.690, *β* = 0.119, *p* = 0.018) were significant predictors, indicating that poor sleep quality adversely affects empathy. Additionally, caregiving duration (*B* = − 4.448, *β* = − 0.325, *p* < 0.001) showed a consistent negative effect, reflecting the toll of long-term caregiving on empathic tendencies (Table 6). These findings highlight the role of sociodemographic factors, caregiving conditions, and sleep quality in shaping empathic tendencies among caregivers, emphasizing the need for tailored interventions to support caregivers’ well-being.

**Table 6:** Emphatic Tendency Scale hierarchical linear regression analysis

	**Model 1**				**Model 2**			
	***B***	***SE B***	***β***	***p***	***B***	***SE B***	***β***	***p***
Age	− 0.766	0.123	− 0.294	< 0.001	− 0.940	0.082	− 0.361	< 0.001
Education status	− 0.700	0.877	− 0.042	0.426	− 0.790	0.611	− 0.047	0.197
Perceived economic status	− 0.841	0.961	− 0.037	0.382	1.535	0.510	0.067	0.003
Status of living with the patient	21.968	2.262	0.422	< 0.001	7.870	1.458	0.151	< 0.001
Presence of other dependents (providing care simultaneously to multiple individuals)	12.163	2.085	0.301	< 0.001	− 5.274	1.293	− 0.130	< 0.001
Duration of care	− 4.000	0.696	− 0.292	< 0.001	− 4.448	0.501	− 0.325	< 0.001
Sleep latency					− 8.825	0.776	− 0.407	< 0.001
Sleep duration					1.690	2.614	0.019	0.518
Habitual sleep activity					− 10.721	0.743	− 0.372	< 0.001
Sleep disorder					− 0.799	1.120	− 0.022	0.476
Sleep medication use					− 0.863	1.193	− 0.019	0.470
*R*	.784^a^			< 0.001	.948^a^			< 0.001
*R* square	0.614				0.899			
Adjusted *R* square	0.607				0.896			
Durbin-Watson	2.654				2.346			

*B* unstandardized coefficients, *Std error* standard error, *Beta* standardized coefficients, *R*^*2*^ determination coefficient, *Adj. R*^*2*^ adjusted *R*-squared; *p* < 0.005

## Discussion

The present study aims to clarify the strength and nature of the relationship between the empathic tendencies and sleep quality of caregivers. The findings of the study revealed that there was a strong and negative correlation between empathic tendencies and sleep quality. This suggests that the sleep quality of caregivers increases significantly with an increase in empathic tendencies.

In the study, it was determined that the caregivers had high empathic tendencies. In line with this finding, in the study conducted by Maximiano-Barreto et al. [33] (*n* = 158) examining different levels of empathy among caregivers, it was stated that domestic relationships may have a positive effect on (and elevate) empathy. In a cross-sectional study by Hussien et al. [30] investigating emotional empathy, burden, and depression levels in caregivers (*n* = 186), it was emphasized that caregivers with high emotional empathy showed milder depressive symptoms compared to others. Moreover, caregivers with high empathic tendencies were shown to communicate more effectively with care recipients and better understand their emotional needs during the care process [31, 34]. In another study, it was observed that caregivers with high empathic tendencies provided quiet moments of comfort to patients, which allowed them to better understand their feelings [35]. In a study conducted by Özgünay et al. [36] on caregivers, it was reported that high empathic tendencies of caregivers significantly helped patients to participate more in the treatment process. On the other hand, Jütten et al. [18] examined the cognitive and emotional components of empathy separately due to its complex nature and reported that there is a negative relationship between cognitive empathy and depression, and a positive relationship between emotional empathy and anxiety in caregivers. In a longitudinal study conducted by Kieboom et al. [37] (*n* = 201), it was stated that caregivers with more emotional empathy displayed poorer physical health. However, these results may emphasize the importance of careful evaluation of the empathic tendencies of caregivers and the significance of training programs on this subject.

Another finding of the study was that 42.8% of caregivers had poor sleep quality with a mean score of 8.58 (*SD* = 1.16). Previous studies have reported that caregivers experience significantly more sleep problems compared to the general population [6, 38]. In a cross-sectional study conducted by Babkair et al. [39] with 100 stroke patients and 80 caregivers, it was reported that 46% of the caregivers had poor sleep quality. In another study conducted by Adegbohun et al. [40] examining sleep quality, care burden, and psychological distress in caregivers (*n* = 64), it was emphasized that 31.3% of the caregivers had poor sleep quality. Moreover, a recent systematic review (*n* = 10) reported that at least 72% of caregivers of advanced cancer patients reported moderate or severe sleep disorders [15]. The results of studies conducted with caregivers of cancer patients in Türkiye revealed similar findings [41, 42]. This may be the consequence of physical and emotional burdens on caregivers.

The present study also found a strong negative correlation between empathic tendencies and sleep quality. This indicates that individuals with high empathic tendency scores have lower PSQI scores (i.e., better sleep quality). This suggests that individuals with high empathy levels can manage the emotional demands of the caregiving process more effectively, which improves their general well-being. It was also revealed that caregivers with high empathic tendencies experienced decreased time to fall asleep, increased sleep duration, and reduced levels of daytime dysfunction. Previous studies have emphasized that individuals’ ability to regulate their basic emotional processes is strongly related to sleep quality [35, 43]. Guadagni et al. [28] reported that emotional empathy was associated with good sleep quality. In the study conducted by Tamm et al. [44] examining the effect of sleep restriction on empathy towards pain, it was observed that age-related sleep deprivation played a significant role on empathy and other regulatory processes. Furthermore, while emotional empathy is positively associated with good sleep quality, increased empathy under stress may lead to anxiety, especially in the absence of adequate coping mechanisms. This dual relationship suggests that emotional empathy may be beneficial when well regulated, but may be detrimental when caregivers experience overwhelming stress [45, 46]. Although the study’s findings are consistent with the literature, differences across studies may be due to subjective and objective measures of sleep quality. While subjective measurements are based on individuals’ own perceptions, objective measurements (e.g., polysomnography or actigraphy) can more accurately assess sleep duration and physiological sleep cycles [48]. These differences suggest that the relationship between empathy and sleep quality should be examined in greater depth.

It was also found in the present study that the empathic tendencies of the caregivers decreased as the age of the patient and the duration of care increased. This finding suggests that long-term caregiving and caring for elderly patients may reduce caregivers’ empathy levels, possibly due to emotional exhaustion or elevated stress levels. This suggests that long-term caregivers’ emotional resources are depleted over time, which may negatively affect their empathy levels. Previous studies reported that long-term caregiving to cancer patients is associated with physical and emotional exhaustion and that caregivers experience higher levels of burnout [44, 49]. Therefore, it can be said that this may lead to a decrease in caregivers’ resilience in the face of increasing levels of stress over time and empathic exhaustion. In a study conducted by Blom et al. [50] with caregivers of cancer patients (*n* = 92), it was highlighted that emotional exhaustion and care satisfaction in caregivers had a significant effect on their quality of life. In another study, caregivers of late-stage cancer patients reported that caring for their loved ones was both satisfying and very stressful at the same time [51]. In previous studies conducted in Türkiye, it has been reported that advancing patient age increases the emotional burden on caregivers [52, 53]. These findings may emphasize the importance of emotional support and training programs for caregivers to maintain a consistent and compassionate approach to care.

The present study reveals that caregivers’ empathic tendencies are associated with their sociodemographic factors (e.g., age, perceived economic status) and care conditions (e.g., number of dependents, living with the patient). Furthermore, the study found that sleep quality and duration of care have a significant impact on empathic tendencies. These findings are in line with the related literature [12, 18]. The findings suggest that interventions to improve caregivers’ well-being and empathy levels should be tailored to their sociodemographic factors and individual needs. Specifically, addressing multiple caregiving responsibilities and sleep disorders may enhance caregivers’ quality of life and effectiveness in care processes. For instance, younger caregivers may benefit more from stress management training, while older caregivers may require additional interventions to support their physical health and improve their sleep quality [29].

### Limitations

Despite its strong aspects, the present study also involves certain limitations. The study sample consisted of caregivers that attended a single health facility in a specific geographical region. Therefore, the findings should be interpreted with caution and the applicability of the results should be tested with caregivers from regions with different demographics to ensure generalizability. Self-report questionnaires were used in the study, which may affect the accuracy of the data. Additionally, since the study did not demonstrate cause-and-effect relationships, precise information regarding the direction of the correlation between empathy and sleep quality or how this relationship changes over time may not be obtained. Finally, other psychological and environmental factors (e.g., stress level, living conditions) that may affect the empathic tendencies and sleep quality of caregivers could not be fully tested, which may impact the evaluation of these results. Therefore, it is recommended that future studies utilize larger and more diverse samples, conduct long-term follow-up studies, and carefully select different data collection methods.

## Conclusion

This study found that caregivers generally exhibit high empathic tendencies and good sleep quality, with a significant relationship between the two. Sleep latency and habitual sleep activity emerged as significant predictors of empathy, influenced by sleep quality and various demographic and social factors. Notably, interventions targeting sleep quality could enhance caregivers’ empathic abilities by 28.9%. A multidisciplinary approach is essential to support caregivers in maintaining healthy empathy and consistent care. For example, interventions such as empathy training programs and sleep hygiene education may be effective in both increasing empathic tendencies and supporting physical and psychological well-being. Individual counseling or group-based programs to manage sleep disorders may help caregivers maintain their capacity for empathy by improving sleep quality. In addition, psychoeducational programs and social support groups can help caregivers healthily manage their empathic abilities. These interventions reduce the risk of burnout in caregivers and provide a more sustainable caregiving process that enhances the well-being of patients. Future research should assess the effectiveness of such interventions and examine the long-term effects of caregiving on empathy and overall health.

## Data Availability

The data supporting the findings of this study are available from the corresponding author upon reasonable request.
